# Glutathione S-transferase Pi expression predicts response to adjuvant chemotherapy for stage C colon cancer: a matched historical control study

**DOI:** 10.1186/1471-2407-12-196

**Published:** 2012-05-28

**Authors:** Lucy Jankova, Graham Robertson, Charles Chan, King L Tan, Maija Kohonen-Corish, Caroline L-S Fung, Candice Clarke, Betty P C Lin, Mark Molloy, Pierre H Chapuis, Les Bokey, Owen F Dent, Stephen J Clarke

**Affiliations:** 1 Cancer Pharmacology Unit, ANZAC Research Institute, Concord Hospital, The University of Sydney, Sydney, NSW, 2139, Australia; 2 Department of Anatomical Pathology, Concord Hospital and Discipline of Pathology, The University of Sydney, Sydney, NSW, 2139, Australia; 3 Department of Anatomical Pathology, Concord Hospital, Sydney, NSW, 2139, Australia; 4 Cancer Research Program, Garvan Institute of Medical Research and St Vincent’s Clinical School, University of New South Wales, Sydney, NSW, 2052, Australia; 5 Australian Proteome Analysis Facility and Department of Chemistry and Biomolecular Sciences, Macquarie University, Sydney, NSW, 2109, Australia; 6 Department of Colorectal Surgery, Concord Hospital and Discipline of Surgery, The University of Sydney, Sydney, NSW, 2139, Australia; 7 Department of Medicine, Concord Hospital and Discipline of Medicine, The University of Sydney, Sydney, NSW, 2139, Australia

**Keywords:** Colon cancer, GST Pi, Adjuvant chemotherapy, Survival

## Abstract

**Background:**

This study examined the association between overall survival and Glutathione S-transferase Pi (GST Pi) expression and genetic polymorphism in stage C colon cancer patients after resection alone versus resection plus 5-fluourouracil-based adjuvant chemotherapy.

**Methods:**

Patients were drawn from a hospital registry of colorectal cancer resections. Those receiving chemotherapy after it was introduced in 1992 were compared with an age and sex matched control group from the preceding period. GST Pi expression was assessed by immunohistochemistry. Overall survival was analysed by the Kaplan-Meier method and Cox regression.

**Results:**

From an initial 104 patients treated with chemotherapy and 104 matched controls, 26 were excluded because of non-informative immunohistochemistry, leaving 95 in the treated group and 87 controls. Survival did not differ significantly among patients with low GST Pi who did or did not receive chemotherapy and those with high GST Pi who received chemotherapy (lowest pair-wise p = 0.11) whereas patients with high GST Pi who did not receive chemotherapy experienced markedly poorer survival than any of the other three groups (all pair-wise p <0.01). This result was unaffected by GST Pi genotype.

**Conclusion:**

Stage C colon cancer patients with low GST Pi did not benefit from 5-fluourouracil-based adjuvant chemotherapy whereas those with high GST Pi did.

## Background

Glutathione S-transferase Pi (GST Pi) is found in the cell nucleus, cytoplasm and mitochondria of a wide range of normal and neoplastic tissues and is expressed in colorectal cancer [1,2]. Elevated GST Pi is associated with poor prognosis in many cancers, including colorectal cancer [3,4]. Previously we have shown a high level of expression of GST Pi to be associated with adverse histological features and diminished overall survival after adjustment for other prognostic factors in a large series of patients who had a potentially curative resection for stage C colonic adenocarcinoma [5]. That study and many others in colorectal cancer have been prognostic rather than predictive in that they have examined GST Pi in consecutive patients who have received the same treatment or mix of treatments, without random allocation to different treatment groups. Predictive biomarkers, on the other hand, can indicate the likely effect of specific adjuvant treatment on patient outcomes such as tumour recurrence or survival.

In colorectal cancer, the prognostic or predictive value of GST Pi expression or genomic polymorphisms for outcomes after chemotherapy has been examined in several studies. However, in most cases, the patients had received palliative treatment for advanced metastatic disease rather than adjuvant treatment in earlier stages and in most reports there was no random allocation to different treatments. Overall, there is some evidence for GST Pi as a prognostic biomarker but little evidence for its predictive ability.

As far as we are aware, there has not yet been any attempt to determine whether GST Pi expression or genotype can predict response to adjuvant chemotherapy in stage III patients with colon cancer, yet this is the group of patients for whom adjuvant chemotherapy has most clearly been demonstrated as beneficial in randomised controlled trials [6]. If biomarkers able to predict the results of chemotherapy were found, this could lead to more accurate targeting of treatment to those stage C patients most likely to benefit and to avoidance of inappropriate treatment, with its costs and possible toxicity, in those unlikely gain any advantage.

The ability of a tumour marker to predict response to chemotherapy cannot be determined from a patient series in which some patients were specifically selected for chemotherapy and others were not. At first glance one might simply search for a statistical interaction between the marker and chemotherapy by comparing outcome in four categories of patients: those with a low (or negative) value of the marker who did versus did not receive chemotherapy and those with a high (or positive) value who did versus did not receive chemotherapy. However this would fail to take account of the factors leading to selection or rejection for chemotherapy, which are themselves likely to be associated with survival potential. Comorbidity and advanced tumour stage, for example, are likely to militate against treatment but also to be associated with diminished survival. The ideal research design for identifying the predictive ability of a marker would certainly involve searching for a statistical interaction effect among the four groups defined above, but the patients would need to have been randomly allocated to chemotherapy treatment versus surgery alone [7]. For ethical reasons, because chemotherapy is currently given routinely to a large proportion of stage C colon cancer patients, it is extremely unlikely that such a randomised trial could now be conducted. An alternative would be to use data from a historical randomised trial with the addition of results for a marker obtained by retrospective histopathological assessment of archived tissue, although this approach is very rare. An example of such a research design is the report by McLornan et al., although that study showed no significant effect from chemotherapy [8].

Using a prospective hospital-based registry of resections for colorectal cancer we were able to construct a historical control group of patients who had a resection for stage C colonic cancer before chemotherapy was introduced in our hospital in April 1992 and who could be matched with patients from May 1992 to December 2004 who did receive chemotherapy. We acknowledge that this is less than ideal but it is a reasonable surrogate design which can yield legitimate information about the interaction between GST Pi and 5-fluourouracil (5-FU) based chemotherapy in relation to overall survival and it is a feasible design which could be used by others with access to a large historical database and archival tissue bank for colorectal cancer.

The aim of this study was to examine the association between GST Pi expression and overall survival in patients who had received adjuvant chemotherapy after resection of a stage C colon cancer between May 1992 and December 2004 and an age- and sex-matched historical control group of equivalent patients who had been treated by surgery alone before the introduction of adjuvant chemotherapy. A secondary aim was to examine the survival of these patients according to GST Pi genotype.

## Results

Between May 1 1992, when adjuvant chemotherapy was introduced in this hospital, and December 31 2004 resections for stage C colon cancer were performed on 263 patients, of whom 104 (40%) received adjuvant chemotherapy. The proportion receiving chemotherapy increased steadily from 11% in 1992 to 71% in 2004. One hundred and four age- and sex-matched controls were selected from among patients who had a resection for colon cancer between January 1979 and April 1992. Of the total of 208 patients, 26 had insufficient archived tissue for immunohistochemical evaluation, leaving 182; comprising 95 in the chemotherapy group and 87 in the control group. Fifty-two percent in both groups were male and mean ages were 64.1 years (SD 9.5) in the chemotherapy group and 63.6 (SD 10.6) in the control group (Table 1). There was no significant difference between the two groups on any of the 10 pathological characteristics examined in Table 1.

**Table 1:** Background characteristics and tumour pathology in patients who received adjuvant chemotherapy and in age/sex-matched historical controls

	Chemotherapy Group N = 95	Control group N = 87	Chi^2^ p
Age (mean, SD)	64.1 (9.5)	63.6 (10.6)	--
Male	49 (52)	45 (52)	--
Tumour site right colon	50 (53)	40 (46)	0.37
Size ≥ 5 cm	43 (45)	43 (49)	0.57
Tumour spread beyond muscularis propria	84 (88)	80 (92)	0.43
Mucinous or signet ring adenocarcinoma	16 (17)	10 (12)	0.30
≥ 4 lymph nodes involved	26 (27)	26 (30)	0.70
Apical node involved	11 (12)	11 (13)	0.83
High grade tumour	34 (36)	31 (36)	0.98
Venous invasion present	20 (21)	23 (26)	0.39
Free serosal surface involved	22 (23)	23 (26)	0.61
Adjacent structure infiltrated	6 (6)	4 (5)	0.75*

*Fisher’s exact test.

Number (%).

In our earlier detailed study of the prognostic value of GST Pi we showed that the percentage of cells with nuclear staining in the central part of the tumour was the single best measure of GST Pi expression as an independent predictor of overall survival, the optimum cutting point for defining high versus low GST Pi being ≤ 40% versus >40% [5]. At this cutting point in the present study high GST Pi occurred in 63 out of 182 patients (35%). In the chemotherapy group 40% of patients had high GST Pi as compared with 29% in the control group, the difference being non-significant (p = 0.11).

The policy of the database is to follow patients yearly for up to 14 completed years. At the time of analysis, 98 patients had died, 3 had been lost to follow-up, 24 had completed 14 years of follow-up and follow-up was continuing for 57. In patients who had not died (including those lost) follow-up time ranged from 50 months to 280 months with a median of 126 months.

**Figure 1:**
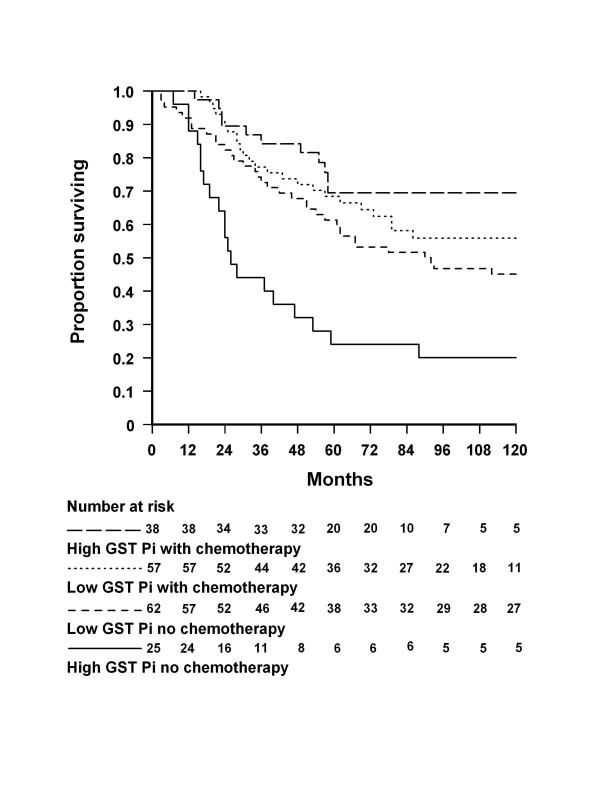
**Overall survival by patient group and GST Pi expression.** Overall survival in four patient groups: those with high GST Pi who received adjuvant chemotherapy, those with low GST Pi who received chemotherapy, those with low GST Pi who did not receive chemotherapy, and those with high GST Pi who did not receive chemotherapy. Differences between the first three groups were not statistically significant (lowest p = 0.11) whereas differences between all of those groups and the fourth group were significant (all p < 0.01)

In the overall pool of 182 patients, survival was significantly poorer in those who had not received chemotherapy than in those who had (hazard ratio [HR] 1.8, 95% confidence interval [CI] 1.2–2.8, p = 0.005). It would not be meaningful to draw a similar overall comparison between patients with low and high GST Pi because of the potential for chemotherapy to attenuate the association between GST Pi and survival in the chemotherapy group. However, in patients who had not received chemotherapy, overall survival was significantly poorer among those with high central nuclear GST Pi than among those with low GST Pi (HR 2.3, CI 1.4–3.9, p = 0.002). Analysis of survival stratified by both high versus low GST Pi and chemotherapy versus no chemotherapy showed no significant difference among patients with low GST Pi who did or did not have chemotherapy and those with high GST Pi who had chemotherapy (lowest p = 0.11) whereas patients with high GST Pi who did not have chemotherapy experienced significantly poorer survival than any of the other groups (all p < 0.01) (Table 2, Figure 1). This was corroborated by Cox regression modelling where high GST Pi and absence of chemotherapy were coded 2 and the alternatives were coded 1. The product of these two variables showed a statistically significant interaction (p = 0.008). Furthermore the interaction term remained statistically significant (p < 0.001) when it was included in a model with all variables in Table 1 that had a bivariate p value of ≤ 0.1 with overall survival (Table 3). Thus, while low GST Pi was not associated with any difference in response to chemotherapy, high GST Pi in the absence of chemotherapy predicted a markedly poorer outcome than might otherwise be expected. The implication is that patients with low GST Pi did not benefit from 5FU-based chemotherapy whereas those with high GST Pi clearly did.

**Table 2:** Five-year overall Kaplan-Meier survival rates for stratified analysis of chemotherapy group versus control group by high versus low nuclear GST Pi expression in central tumour tissue

	5-year survival rate (95% confidence interval)
Low GST Pi with chemotherapy	68 (55–79)
Low GST Pi without chemotherapy	61 (48–72)
High GST Pi with chemotherapy	69 (52–82)
High GST Pi without chemotherapy	24 (10–42)

**Table 3:** Overall survival for sex, age, pathology features and GST Pi by chemotherapy interaction

	Bivariate HR*	Bivariate p value	Multivariable HR (95% CI)	Multivariable p value
GST Pi by chemotherapy interaction	--	--	1.4 (1.2–1.8)	<0.001
Male sex	1.0	0.850	--	--
Age ≥ 75 years	1.7	0.044	1.5 (0.9–2.6)	0.153
Right colon tumor	0.7	0.063	0.8 (0.5–1.3)	0.431
Size ≥ 5 cm	1.3	0.193	--	--
Spread beyond muscularis propria	3.0	0.030	1.6 (0.6–4.6)	0.356
Mucinous or signet ring	1.1	0.730	--	--
≥ 4 lymph nodes involved	2.5	<0.001	1.5 (0.97–2.4)	0.071
Apical node involved	2.8	<0.001	2.0 (1.1–3.5)	0.021
High grade tumor	2.3	<0.001	1.7 (1.1–2.7)	0.016
Venous invasion present	1.5	0.084	1.2 (0.8–1.9)	0.418
Free serosal surface involved	3.3	<0.001	2.0 (1.2–3.1)	0.004
Adjacent structure infiltrated	1.3	0.455	--	--

* Hazard ratio.

The determination of genotype was uninformative in 13 specimens, leaving 169 for analysis; 81 (48%) having the Ile/Ile genotype, 73 (43%) the Ile/Val genotype and 15 (9%) the Val/Val genotype, which was similar to other studies [9-11]. The frequency of high GST Pi did not differ significantly by genotype (Ile/Ile 41%, Ile/Val 29%, Val/Val 33%; p = 0.295) and there was no difference in the proportions of patients receiving chemotherapy (Ile/Ile 44%, Ile/Val 51%, Val/Val 40%; p = 0.637). There was no overall association between genotype and survival (all pair-wise p > 0.15) and no association between genotype and survival in the chemotherapy group alone (all pair-wise p > 0.42) or in the control group alone (all pair-wise p > 0.13). When the joint association between chemotherapy, GST Pi and survival shown in Figure 1 was further stratified by genotype it was again found that, for patients with the Ile/Ile genotype, those with high GST Pi who did not receive chemotherapy had significantly poorer survival than the other three groups (all pair-wise p <0.003) and there were no significant differences among those three groups (all pair-wise p > 0.385). The same was true for the Val/Val genotype (the corresponding pair-wise p values were p <0.046 and > 0.527) and there was a non-significant tendency towards the same pattern for the Ile/Val genotype, where there were only nine patients in the high GST Pi/no chemotherapy group. From these results it was concluded that genotype had no influence on the association between survival and the joint effects of chemotherapy and GST Pi expression.

## Discussion

GST Pi is a member of the glutathione-S-transferase superfamily of phase II xenobiotic-metabolizing enzymes that catalyse the conjugation of endogenous and exogenous electrophiles, including reactive oxygen species, toxins, carcinogens and anti-cancer agents, to the nucleophilic thiol group of reduced glutathione (GSH) [12]. A number of studies have concentrated on the connection between aberrant expression of GST isozymes, including GST Pi isozymes, with the development and expression of resistance to chemotherapy drugs as reviewed by McIlwain *et al.*[13]. From this perspective an expected result should have been a poor response to chemotherapy in patients with high expression of GST Pi. However our study has shown that, for stage C colon cancer patients, overall survival was significantly and markedly poorer in patients with high GST Pi who did not receive chemotherapy than in those with high GST Pi who did. Furthermore, survival in the latter group was no different from that in patients with low GST Pi, whether or not they received chemotherapy. Expressed differently, chemotherapy had no apparent effect on survival in patients with low GST Pi whereas those with high GST Pi appeared to benefit considerably. This finding could be explained by the fact that a major function of GST Pi is not only the phase II metabolism of drugs but to also contribute to the detoxification of endogenously generated reactive oxygen species and the associated potentially toxic macromolecules and lipid peroxides produced in cells. An example of this detoxificant/antioxidant role is the induction of GST Pi as part of the coordinated defence mechanism to protect colonic cells against oxidative injury [14]. The presence of high GST Pi in colon tumours may confer a selective advantage on malignant colon cells, as increased production of reactive oxygen species is a feature of cancer progression [15]. Therefore the poorer overall survival in patients with high GST Pi who did not receive chemotherapy could be attributed to the ability of GST Pi to counter the elevated oxidative stress, enhancing persistence of malignant colon cells and thereby leading to poorer survival. This is also consistent with our previous finding of increased GST Pi expression being an independent prognostic factor for reduced overall survival [5]. Furthermore, treatment with diverse chemotherapy drugs tips the redox balance of already stressed cancer cells and elicits selective additional toxicity that ultimately kills them. In this regard the recent reports of the ability of 5-FU to promote oxidative stress in colonic cancer cells [16,17], in addition to its multiple actions as an anti-metabolite, including inhibition of thymidylate synthase, may provide an explanation for the clear benefit of 5-FU based treatment in those patients with high GST Pi (Figure 1). The additional generation of reactive oxygen species by 5-FU may overwhelm the advantage of high GST Pi in colon cancer cells either by directly increasing the molecular species detoxified by GST Pi enzyme activity and/or by depletion of the available pool of GSH required for conjugation reactions performed by GST enzymes. GSH is also the major anti-oxidant used by cells in non-enzymatic reactions to protect against reactive oxygen species.

Accordingly the implication of our finding is that stage C colon cancer patients with low intracellular concentrations of GST Pi may not need to be treated with 5-FU-based adjuvant chemotherapy whereas those with high GST Pi definitely should be treated. As far as we are aware, this is the first study to demonstrate the predictive value of GST Pi expression in regard to chemotherapy for stage C colon cancer.

A secondary finding was that GST Pi genotype had no apparent influence on survival or on the association between survival and the joint effects of GST Pi expression and adjuvant chemotherapy. Interest in the effect of GST Pi polymorphisms arose from the concept that variant GST Pi proteins may differentially affect the actions of chemotherapeutic drugs. The single nucleotide polymorphism A313G in exon 5 results in a valine for isoleucine substitution producing a Val/Val variant with reduced GST Pi enzyme activity [18,19]. In metastatic colorectal cancer Stoehlmacher *et al*. found poorer overall survival in patients with the Ile/Ile genotype than in those with the Ile/Val or Val/Val genotypes after combined 5-FU/oxaliplatin chemotherapy [9,20] although this was not replicated by other researchers using the same agents [21]. Also in 5-FU/oxaliplatin-treated patients poorer survival was seen in those with the Ile/Ile and Ile/Val genotypes compared with the Val/Val genotype [22]. Similarly, significantly poorer survival associated with the Ile/Ile genotype as compared with the Ile/Val or Val/Val genotypes was reported in patients treated with FOLFOX-4 [10], although two other studies using FOLFOX showed no difference in survival between genotypes [23,24]. Additionally, a study of oxaliplatin treatment [25] and two others using a mixture of agents and regimens showed no differences between GST Pi genotypes [11,26]. All of the above were prognostic studies as all patients had received chemotherapy and there was no random allocation to different treatment groups. A truly predictive study that randomly allocated patients to one of three 5-FU-based treatment strategies also showed no differences between GST Pi genotypes [27] while another study with patients randomised to first-line capecitabine alone versus capecitabine plus irinotecan showed a short, but significant improvement in progression-free survival for the Val/Val genotype only [28]. Two remaining studies included patients with colorectal tumours of all Dukes/TNM stages, not just those with metastatic disease, but produced contradictory results. Holley et al. found no association between GST Pi genotype and overall survival [29] whereas Jones et al. found that the Ile/Ile genotype was associated with decreased survival as compared with the Ile/Val and Val/Val genotypes [30]. Thus the evidence for an association between GST Pi genetic polymorphism and chemotherapeutic treatment and survival (or other patient outcomes) is inconclusive. The reasons may relate to differing study designs; for example retrospective versus prospective, randomized versus non-randomized allocation to treatment; varying stage mixes of patients and hence different mixes of palliative and adjuvant treatment; various chemotherapeutic agents and regimens; and statistical issues such as limited numbers of patients available for analysis. The latter is particularly a problem in relation to the Val/Val genotype which has a prevalence of only 8% to 10% which may lead to type II errors in small patient samples.

A limitation of the present study is that patients were not randomly allocated to the treatment and control groups. Instead the treatment group comprised all patients selected for chemotherapy since April 1992 when it became available in this hospital, whereas the control patients were drawn from the period from 1979 to April 1992 and individually matched with treated patients on age and sex. However there was no significant difference between the treated group and the control group on 10 other background and tumour pathology characteristics. Comorbidity may have influenced survival and may have differed between the two groups but we were unable to compare them because details of comorbidity were not recorded in the database until 1995. However, as the survival of patients with low GST Pi did not differ significantly between the control and treatment groups, it is most unlikely that differential comorbidity could have accounted for the marked treatment/control survival difference in patients with high GST Pi alone. A further limitation was the relatively small number of patients available for analysis; there were 182 in total and 169 for the genotype analyses, which may have limited our ability to find statistically significant differences between some groups. Nevertheless the markedly poorer survival of the high GST Pi/non-chemotherapy group (Figure 1) suggests that this interaction would be likely to persist in a larger study.

Earlier studies of the association between GST Pi expression and patient outcomes have yielded varying results. A significant independent association between high GST Pi and diminished overall survival was found by Mulder et al. and a similar independent association was found by Sutoh et al. for disease-free survival [3,4]; both studies included colonic and rectal tumours and all four Astler Coller or Dukes stages. Contrary to these results Kim et al. found no association between GST Pi expression and overall survival in patients with stage IV colorectal cancer, all of whom had been treated by 5-FU/oxaliplatin palliative chemotherapy [21]. Given that tumour stage is the most clearly established prognostic variable in colorectal cancer it is reasonable to expect that other prognostic factors, including biomarkers, may behave differently within different stages and also that they may behave differently between the colon and rectum or even at different sub-sites. In our study we focussed on stage C colonic tumours because of the known beneficial effects of chemotherapy in this patient group and the desire to find biomarkers which would allow more refined targeting of patients most likely to benefit from chemotherapy and to avoid costly and possibly toxic treatment of those who are unlikely to benefit.

## Conclusions

Survival did not differ between patients with low GST Pi, whether or not they had chemotherapy and those with high GST Pi who had chemotherapy, whereas patients with high GST Pi who did not receive chemotherapy had markedly poorer survival than the other three groups. GST Pi genotype was not associated with survival and did not influence the relationship between GST Pi expression, chemotherapy and survival. Given that adjuvant chemotherapy is currently recommended and routinely used in patients with stage C colon cancer it is now unlikely, for ethical reasons, that a randomised trial including a surgery-only control group could be conducted to confirm our findings. However such a study, based on an early randomized trial and archived tissue, may be possible.

## Methods

Information on patients having a resection for colorectal cancer performed by members of the Concord Hospital Department of Colorectal Surgery has been entered into a prospective computer database since 1971 [31,32]. The data set contains information on patient characteristics, co-morbidity, presentation, investigations, surgical management, complications, adjuvant therapy, pathology and follow-up, and has the approval of the South Western Sydney Health Area Ethics Committee. All patients gave written consent personally or through their guardian for pathology specimens and anonymous clinical data to be used for research purposes. From 1981, all resections were performed by specialist colorectal surgeons according to a standardized procedure [33] and acquisition of clinical data has been conducted by a single surgeon (P.C.). Patients reported here had a resection for clinicopathological stage C colon cancer between 1979 and 2004 inclusive.

### Adjuvant chemotherapy

The chemotherapy regimens utilized involved, for the most part, 6-month courses of bolus injections of 5-FU and folinic acid administered daily for 5 days every 28 days over a total of 6 cycles (Mayo Clinic regimen [34]) or 5-FU and leucovorin repeated weekly for 6 doses with a 2-week rest between (Roswell Park regimen [35]). These regimens were used as they were supported by results from randomized controlled trials on patients with stage C colon cancer.

### Selection of the historical control group

Patients who received adjuvant chemotherapy were matched individually by sex and age with controls selected from among patients who had a resection for stage C colon cancer between 1979 and April 1992, before radiotherapy and chemotherapy were introduced at this hospital [36]. Matching was by sex because numerous studies have shown sex differences in many epidemiological, clinical and pathological characteristics of colorectal cancer [37] and by age because of the demographic association between advancing age and diminishing survival in the population at large.

### Histopathology

Pathological examination of the resected specimen followed a standard protocol as described previously [38]. Only adenocarcinomas (including mucinous and signet ring cell carcinomas) were included in the data set. Where multiple tumours were present, only the lesion with the most advanced stage was included. Tumour size was measured as the greatest surface dimension. Blocks were taken to demonstrate maximum direct tumour penetration of the bowel wall. Additional blocks were taken specifically to demonstrate the relationship between tumour and any adherent structure or tissue [39] as well as lines of resection and the free serosal surface [40]. Venous invasion by tumour referred to involvement of thick or thin walled veins, either within or beyond the bowel wall. When doubt existed as to whether a structure involved was a vein, a negative finding was recorded. Tumour grade was assessed taking into account the degree of differentiation and anaplasia, the nature of the tumour margin (pushing or infiltrating) and the presence and prominence of vascular invasion [31]. An apical lymph node was defined as the most proximal of any nodes found within 1 cm of the ligation of a named vessel as the apex of a pedicle [41]. All pathological characteristics analyzed were looked for in every specimen and their presence or absence recorded explicitly. There were no missing data on any original database variable. Tumours were staged according to the Australian Clinicopathological Staging System for colorectal cancer which accommodates sub-stages compatible with other clinicopathological staging systems such as Tumour Nodes Metastases [42]. A stage C tumour was defined as one with lymph node metastasis but no systemic metastasis and no tumour present in the proximal, distal or deep lines of resection histologically.

### Tissue microarray construction

Tissue micro arrays (TMA) for the assessment of GST Pi were constructed using an Advanced Tissue Arrayer ATA-100 (Chemicon, Temecula, Ca). 1.0 mm cores were taken from carefully selected, morphologically representative areas of the original paraffin blocks and arrayed into freshly made recipient paraffin blocks. As it is known that there is heterogeneity within colorectal cancers, we took cores from (a) the central part of the tumour, avoiding the luminal surface, the tumour edge and areas of necrosis, (b) the deep invasive tumour front at the interface between the tumour and non-neoplastic tissue, and also (c) adjacent normal mucosa.

### Immunohistochemistry

GST Pi (1:20, Abcam, ab17088, Cambridge, U.K.) immunohistochemistry was carried out using DAKO Autostainer (DAKO, Glostrup, Denmark). Following dewaxing and rehydration, antigen retrieval was performed in a water bath (95°C) for 30 minutes using sodium citrate (pH 6.0) Target Retrieval Solution S1699 (DAKO, Glostrup, Denmark). Endogenous peroxidases were blocked with 3% hydrogen peroxide for 5 minutes. Non-specific binding sites were blocked with Protein Block (DAKO, Glostrup, Denmark) for 10 minutes. The sections were incubated with diluted GST Pi antibody for 1 hour at room temperature, followed by secondary reagent EnVision + Dual Link System-HRP (DAB+) K4065 (DAKO, Glostrup, Denmark) for 30 minutes. Staining was completed by a 10 minute incubation with 3,3-diaminobenzidine (DAB+) substrate-chromogen. After buffer wash the slides were counterstained with haematoxylin, dehydrated and mounted.

### Immunohistochemical evaluation

Immunoreactivity for GST Pi was assessed independently by three experienced pathologists (K.T., C.F., C.C.) who were unaware of the patients’ clinical characteristics, other histopathological data and survival. Tissue cores from the central part of the tumour and the invasive front were assessed separately in each sample, as was the presence of nuclear and cytoplasmic staining in the tumour epithelial cells. The intensity of staining was graded as 0 (no staining), 1 (weak staining), 2 (intermediate staining), 3 (strong staining). The percentage of stained cells (hereinafter termed “percentage stained”) was recorded as a quasi-continuous variable coded 0%, 1%, 10%…90%, 100%. When there were discrepancies between the observers, the slides were reviewed and a consensus reached. To find the optimum dichotomy for percentage stained in relation to survival the distribution of percentage stained was first dichotomized at 0% versus 1–100% and survival curves with the associated p value were obtained. The cutting point was then raised in steps of 10% (0–9% vs. 10–100%, 0–20% vs. 30–100% … 0–90% vs. 100%) and the separation of curves and p value recorded at each step. This process yielded the optimum cutting point giving the greatest separation of survival curves [43].

### Genotyping

Archival paraffin block sections of all the lymph nodes resected from each patient were first reviewed by a pathologist (CC), who selected one normal cancer-free lymph node from each patient for subsequent analysis. A core biopsy was taken from the tissue block and DNA was extracted with the Puregene DNA Isolation Kit (Gentra, Minneapolis, MN) as previously described [44,45]. A custom Taqman SNP Genotyping Assay (Applied Biosystems, Foster City, CA) was used for genotyping. The primer and probe sequences for GSTP1 were as follows:

Forward primer 5′-CCTGGTGGACATGGTGAATG-3′;

Reverse primer 5′- TGGTGCAGATGCTCACATAGTTG-3′;

Probe 1 (VIC-labelled) 5′-TGCAAATACATCTCC-3′;

Probe 2 (FAM-labelled) 5′-CTGCAAATACGTCTCC-3′ [27].

The DNA samples were diluted to ~5 ng/ml and tested in triplicate. Each 10μml reaction mix contained 5 μl of Taqman Universal PCR Master Mix (Applied Biosystems), 2 μl of 5x SNP Genotyping Assay, and 3 μl (~15 ng) of DNA. The PCR reactions and SNP analysis were carried out on the ABI 7900 (Applied Biosystems), with PCR conditions as follows: 50°C (2 min); 95°C (10 min); 40 cycles of 95°C (15 s) followed by 60°C (1 min).

### Follow-up and survival

Apart from patients lost to follow-up, all patients were followed annually until death or for up to 14 years or to December 31, 2009. Overall survival time was measured from resection until the date of death due to any cause, the censoring date being the date of last follow-up for those surviving or the date of last contact for those lost to follow-up.

### Statistical analysis

The chi-squared test or Fisher’s exact test were used to examine the statistical significance of differences in proportions. Comparisons of survival time between strata of binary variables were made with the Kaplan-Meier method and log-rank test. Proportional hazards regression and the Wald test were used in multivariable modeling with product terms to identify potential interactions. The assumption of proportional hazards was assessed by examining plots of log cumulative hazard for parallelism and in no case was it materially violated in any variable included in a regression model. The level for two-tailed statistical significance was p# 0.05 with confidence intervals (CI) at the 95% level. Analyses were performed with SPSS 15.0 for Windows (SPSS Inc., Chicago, Il. USA).

## Abbreviations

GST Pi, Glutathione S-transferase Pi; 5FU, 5-fluourouracil; GSH, The nucleophilic thiol group of reduced glutathione.

## Competing interests

None of the authors have any financial or non-financial competing interests in relation to this paper.

## Authors’ contributions

LJ had general oversight of the study, conducted immunohistochemistry, and participated in interpretation of results and writing. GR was involved in the conception and design of the study and interpretation of results of the genotyping. CCh, KT, CF, BL were responsible for the immunohistochemical evaluation and contributed to the conception and design of the study and the interpretation of results. C Cl conducted the tissue microarray construction and preparation of data for analysis. MK conducted the genotyping and interpretation of results and contributed to drafts of the paper. MM contributed to the conception and design of the study and critical review of the paper. PC and LB were responsible for the surgery and oversight of the clinical database and contributed to the conception and design of the study. OD contributed to the conception and design of the study, conducted the statistical analyses and drafted the paper. SC contributed to the conception and design of the study, oversaw the adjuvant chemotherapy and was involved in writing the paper and general critical review of the work. All authors read and approved the final manuscript.

## Pre-publication history

The pre-publication history for this paper can be accessed here:

http://www.biomedcentral.com/1471-2407/12/196/prepub
